# Correction: Green-synthesised cerium oxide nanostructures (CeO_2_-NS) show excellent biocompatibility for phyto-cultures as compared to silver nanostructures (Ag-NS)

**DOI:** 10.1039/d2ra90068h

**Published:** 2022-07-08

**Authors:** Qaisar Maqbool

**Affiliations:** National Institute of Vacuum Science and Technology (NINVAST) NCP Complex Islamabad Pakistan Qaisar.vu@gmail.com; Department of Biotechnology, Virtual University of Pakistan Lahore Pakistan

## Abstract

Correction for ‘Green-synthesised cerium oxide nanostructures (CeO_2_-NS) show excellent biocompatibility for phyto-cultures as compared to silver nanostructures (Ag-NS)’ by Qaisar Maqbool, *RSC Adv.*, 2017, **7**, 56575–56585, https://doi.org/10.1039/c7ra12082f.

The author regrets that Fig. 4 and 5 of the original article did not appropriately represent the findings.

The correct version of Fig. 4 is shown below. In addition, the associated text on page 56578 “Experimental findings show total mass loss…” should be changed to “Experimental findings show total mass loss of 57.53% by CeO_2_-NS and 61.12% by Ag-NS.”

**Fig. 4:**
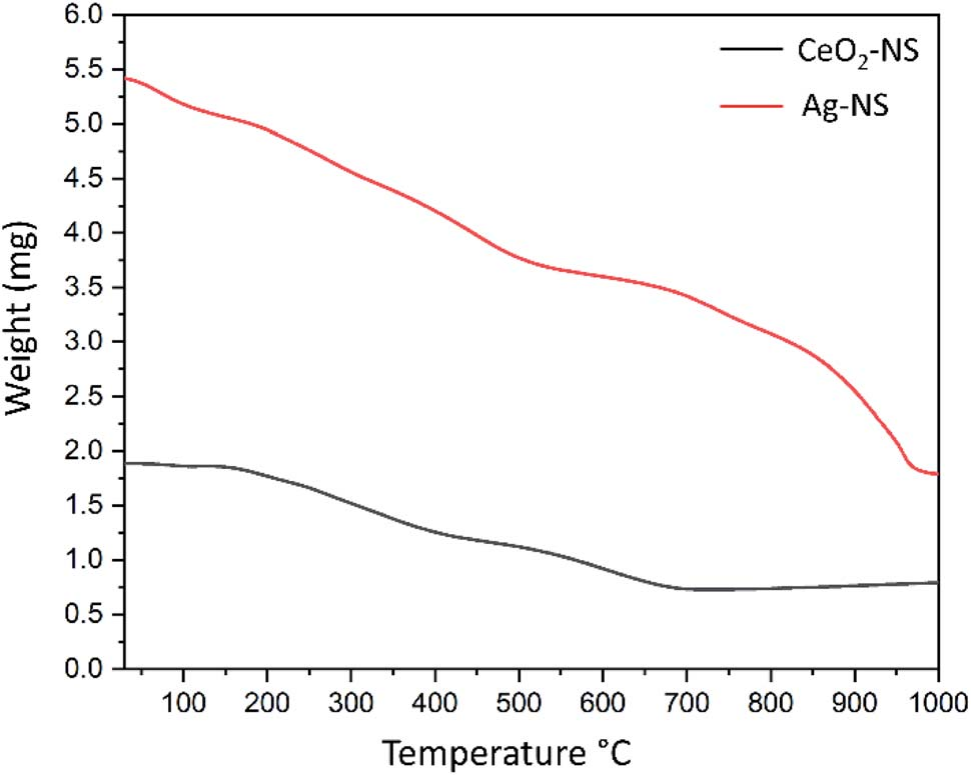
Comparative TGA analysis of CeO_2_-NS and Ag-NS.

Fig. 5 of the original article shows only the plot of selected data points. In order to provide clarity to readers, it should be replaced with the following original FTIR plots (complete scan).

**Fig. 5:**
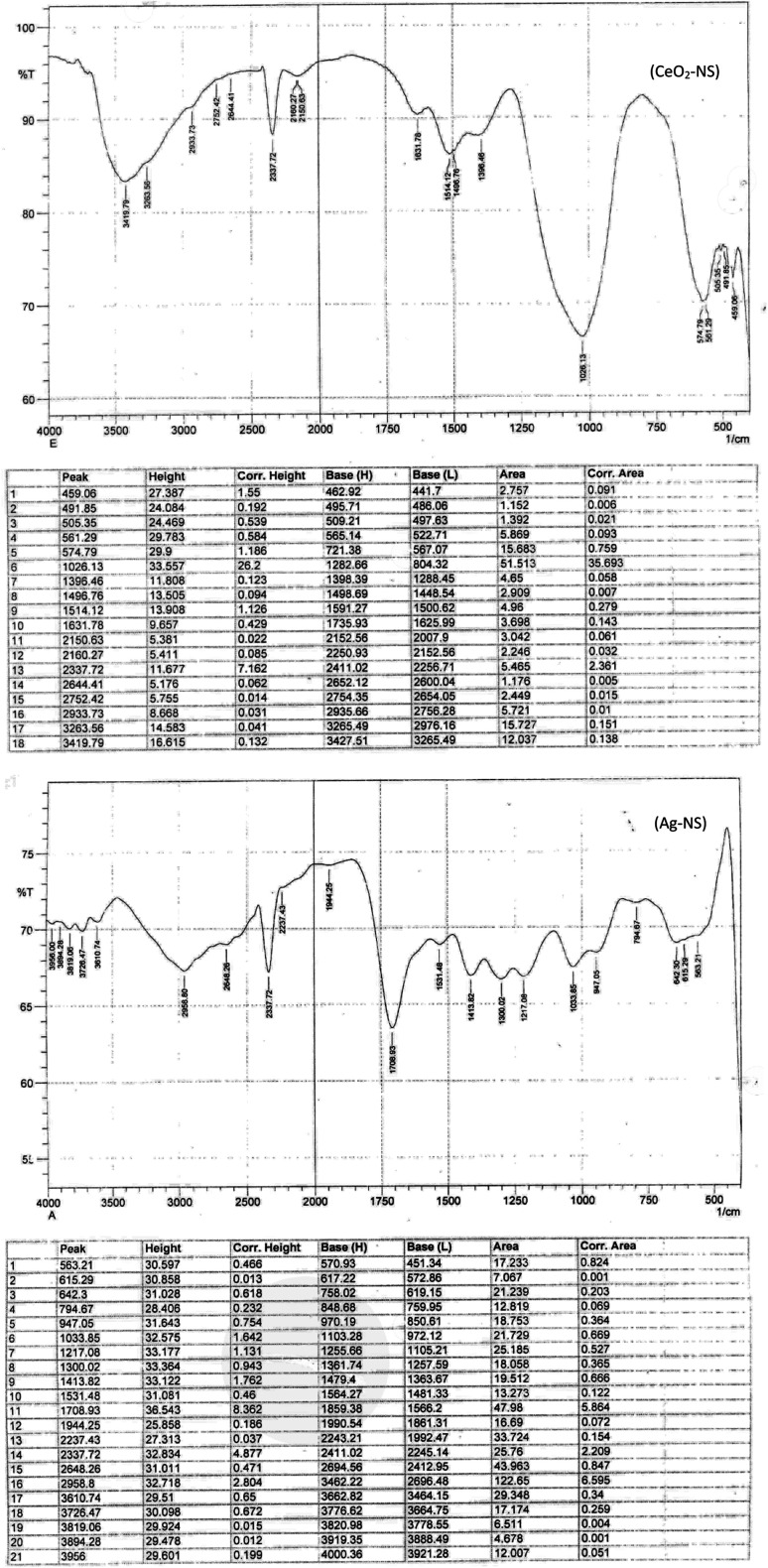
FTIR spectrum of CeO_2_-NS and Ag-NS.

The Royal Society of Chemistry apologises for these errors and any consequent inconvenience to authors and readers.

## Supplementary Material

